# Multimodale Ultraschalltechniken zur Differenzialdiagnose von Milzherden – eine diagnostische Herausforderung

**DOI:** 10.1007/s00104-023-02018-1

**Published:** 2023-11-27

**Authors:** Konrad Friedrich Stock, Julia Slotta-Huspenina, Hajo Findeisen, Christian Görg

**Affiliations:** 1 Internistisches Ultraschall-Labor der Abteilung für Nephrologie, Klinikum rechts der Isar, Technische Universität München, Ismaninger Str. 22, 81675 München, Deutschland; 2 Pathologie Starnberg MVZ GmbH, Starnberg, Deutschland; 3 Klinik für Innere Medizin, Rotes Kreuz Krankenhaus Bremen, Bremen, Deutschland; 4 Interdisziplinäres Ultraschallzentrum, Klinik für Gastroenterologie, Universitätsklinikum Marburg, Marburg, Deutschland; 5 Institut für Pathologie der School of Medicine and Health, Technische Universität München, München, Deutschland

**Keywords:** Milztumor, Ultraschall, Milzbiopsie, Sarkoidose, Lymphom, Splenic tumors, Ultrasonography, Spleen biopsy, Sarcoidosis, Lymphoma

## Abstract

**Hintergrund:**

Milztumoren sind selten und können gerade als bildgebender Zufallsbefund eine differenzialdiagnostische Herausforderung darstellen. Aufgrund fehlender großangelegter Biopsiestudien ist die zur Verfügung stehende Literatur hinsichtlich eindeutiger bildgebender Dignitätskriterien begrenzt.

**Ziel der Arbeit/Fragestellung:**

Die vorliegende Arbeit soll die Chancen einer gezielten ärztlichen Anamneseerhebung sowie die Möglichkeiten und Limitationen der multimodalen Sonographie aufzeigen, um mit einfachen und schonenden Methoden zur richtigen Diagnose bei einem Milzherd zu kommen.

**Material und Methoden:**

Selektive Literaturrecherche und klinische Kasuistiken.

**Ergebnisse:**

Bei der Differenzialdiagnostik fokaler Milzläsionen ist die Information über bestehende hämatoonkologische bzw. inflammatorisch-rheumatologische Vorerkrankungen essenziell, um gerade auch Zufallsbefunde korrekt einzuordnen. Neben B‑Bild-Ultraschall (B-US) und farbkodierter Dopplersonographie (FKDS) liefert vor allem die kontrastverstärkte Sonographie (CEUS) differenzialdiagnostisch entscheidende Hinweise. Während im B‑US echoreiche oder arteriell hypervaskularisierte Milzherde in der FKDS/CEUS meist benigne sind, müssen echoarme und arteriell hypoperfundierte Herde weiter abgeklärt werden. Die ultraschallgesteuerte Milzbiopsie hat zwar ein höheres Blutungsrisiko als die Leberbiopsie, ist aber dennoch die schonendste und effektivste Methode, um bei richtiger Indikationsstellung die histologische Klärung von Milzherden zu erreichen.

**Diskussion/Schlussfolgerung:**

Durch die Kombination aus Anamnese und multimodalen Ultraschallmethoden, ggf. ergänzt durch die sonographisch gesteuerte Biopsie, lassen sich fokale Milzläsionen in den meisten Fällen erfolgreich einteilen mit direkten Auswirkungen auf das weitere klinische Vorgehen.

## Abstract (en)

**Background:**

Splenic tumors are rare and can pose a differential diagnostic challenge, especially as an incidental imaging finding. Due to a lack of large scale biopsy studies the available literature is limited with respect to clear imaging criteria for dignity.

**Objective:**

The present work is intended to show the chances of a targeted elicitation of the medical history as well as the possibilities and limitations of multimodal sonography in order to achieve the correct diagnosis of a splenic lesion using simple and gentle methods.

**Material and methods:**

Selective literature search and clinical case studies.

**Results:**

In the differential diagnostics of focal splenic lesions, information about pre-existing hemato-oncological or inflammatory rheumatological diseases is essential in order to correctly classify incidental findings in particular. In addition to B‑mode ultrasound (B-US) and color-coded Doppler ultrasound (CD-US), contrast-enhanced ultrasound (CEUS) in particular provides crucial differential diagnostic information. While hyperechoic foci in B‑US or arterially hypervascularized splenic foci in CD-US/CEUS are usually benign, hypoechoic and arterially hypoperfused foci in CD-US/CEUS must be further clarified. Although the ultrasound-guided biopsy of the spleen has a higher risk of bleeding than a liver biopsy, it is still the gentlest and most effective method for achieving the histological clarification of splenic lesions when the indications are correct.

**Conclusion:**

Through the combination of the medical history and multimodal ultrasound methods, if necessary supplemented by an ultrasound-guided biopsy, focal splenic lesions can be successfully classified in most cases with a direct impact on further clinical procedures.

Fokale Milzbefunde sind selten. Ihre tatsächliche klinische Bedeutung hängt stark von patientenspezifischen Faktoren ab, weshalb der exakten Kenntnis von Anamnese und Vorbildgebung eine besondere Rolle zukommt. Moderne Ultraschalltechniken erlauben es, erste morphologische Unterscheidungen vorzunehmen. Abhängig von der klinischen Konsequenz dieser Befunde kann – bei einem Teil der Milzherde – eine sonographisch gesteuerte Milzbiopsie ein hilfreiches Werkzeug zur endgültigen, exakten Einordnung sein. Der vorliegende Artikel möchte das Potenzial verschiedener Ultraschalltechniken für die tägliche Differenzialdiagnose von Milzherden kasuistisch beleuchten.

Milzherde sind selten. Bei sonographischer Diagnose liegen in 80 % sekundäre Herdbildungen und in 20 % Zufallsbefunde, sog. Inzidentalome vor [1]. Im Jahr 1999 ergab eine Auswertung von Ultraschalluntersuchungen im fortlaufenden Kollektiv des internistischen Ultraschallzentrums der Universität Marburg (*n* = 200.000) bei ca. 0,3 % (*n* = 550) fokale Milzbefunde. In dem klinisch nicht vorselektionierten Patientengut des Marburger Ultraschall-Labors waren dabei ca. 50 % der Herdbildungen maligne, die größte maligne Subgruppe machte hier das Lymphom aus [1].

Die histologisch gesicherte Studienlage für Milzherde ist – anders als bei der Leber – immer noch dünn, unter anderem bedingt durch die große Zurückhaltung bei der Durchführung von Milzbiopsien aus Angst vor möglichen Komplikationen [2].

In einer kürzlich erschienenen Publikation analysierten Safai Zadeh et al. ein aktuelles Kollektiv (2004–2021) von 139 Patienten mit zufällig diagnostizierten fokalen Milzläsionen, das mit Histologien (*n* = 18) bzw. dem klinisch-radiologischen Verlauf (*n* = 121) korreliert wurde: Es zeigte sich bei nur 9/139 Fällen (ca. 6,5 %) ein Malignom, bei 130/139 Fällen (93,5 %) fanden sich im Patientengut benigne Befunde [3]. Für die wissenschaftliche Auswertung der zufällig entdeckten fokalen Milzläsionen wurden diese in die Gruppen „Milzläsionen mit oder ohne (vor-) bekannte maligne Grunderkrankung“ eingeteilt, eine Unterscheidung, die auch klinisch stets berücksichtigt bzw. worüber Klarheit in der Diagnostik angestrebt werden sollte [3, 4].

## Klinische, multimodale Ultraschalluntersuchung der Milz

**Figure 1:**
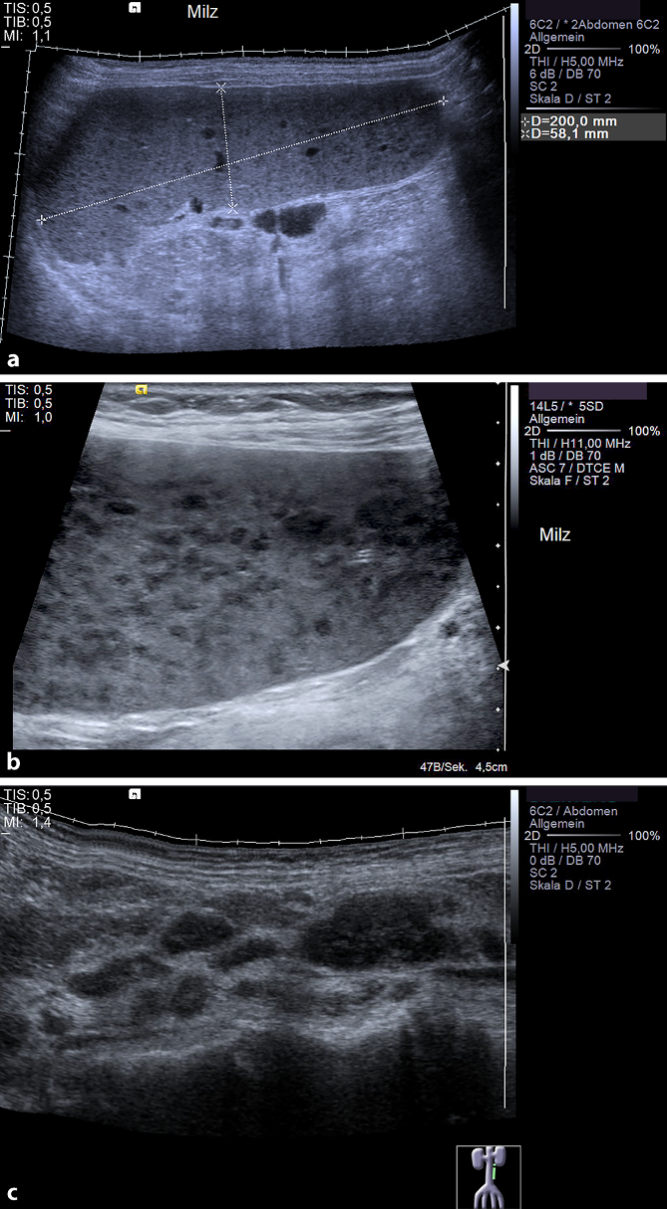

Eine klinische Ultraschalluntersuchung beginnt mit der Anamnese. Neben der expliziten Frage bzw. der klinischen Evaluation einer tumorösen Grunderkrankung müssen zunehmend auch systemisch-infektiologische Ursachen (unter anderem Reiseanamnese, Haustiere) bzw. entzündlich-autoimmune Krankheitsbilder bedacht werden. Bei unklarer Befundkonstellation bzw. entsprechendem klinischem Verdacht auf eine Systemerkrankung ist auch nach der initialen Ultraschalluntersuchung eine weitere Bildgebung bzw. die ergänzende onkologische und rheumatologische Vorstellung anzustreben. Für maligne tumoröse Entitäten gilt: Ein isolierter Milzbefall (z. B. primäres splenisches Lymphom, primäres Angiosarkom der Milz) ist zwar grundsätzlich möglich, jedoch selten, weshalb die Diagnostik stets um eine komplette Abdomensonographie erweitert werden sollte, um potenzielle pathologische Veränderungen anderer Bauchorgane bzw. der Lymphknoten mit zu erfassen (Abb. 1c). Wichtig ist zu wissen, dass ein sekundärer Befall bei ca. einem Drittel aller Patienten mit Morbus Hodgkin und 30–40 % aller Patienten mit Non-Hodgkin-Lymphomen auftritt. Die Häufigkeit eines primären splenischen Lymphoms beträgt nur ca. 1–2 %. Bei Patienten mit bestehender Karzinomerkrankung stellt eine isolierte Milzmetastasierung eine Rarität dar (ca. 1 %; [2]).

Nach dem Vorliegen sämtlicher Befunde ist die interdisziplinäre Besprechung des Patienten im Tumorboard sinnvoll, auch um interdisziplinär die Entscheidung über eine Milzbiopsie mit eindeutiger klinischer Konsequenz festzulegen und ggf. besondere Maßnahmen zur Materialaufbereitung im Vorfeld einer Biopsie zu klären.

### Ultraschalluntersuchungstechnik

Die eigentliche Ultraschalluntersuchung beginnt im *B‑Bild-Ultraschall (B-US)* mit der Darstellung der Milz in deren maximaler Ausdehnung unter Abbildung eines Hilusgefäßes („Milzhilusebene“), sowie der Darstellung der zweiten Ebene zur Berechnung des orientierenden Milzvolumens nach der Rotationsellipsoidformel (Milzvolumen: Länge × Breite × Tiefe × 0,5). Bei sehr großen Organgrößen kann der Einsatz von Ultraschall-Panorama-Verfahren zur Abbildung der gesamten Milz hilfreich sein (Abb. 1a). Normwerte zur Abschätzung der Milzlänge waren über Jahrzehnte die „4711-Regel“ bzw. die Maximallängen von 13 cm für Männer und 12 cm für Frauen [5].

Aktuelle Daten aus einem Kollektiv hessischer Patienten liefert die Arbeit von Chow et al. Hier wurde bei 1230 gesunden Freiwilligen (Stammzellspender) die Milzlänge und das Milzvolumen sonographisch erfasst und zudem in einer Subkohorte von 75 Patienten im Verlauf vermessen. Die Ergebnisse aus der wissenschaftlichen Publikation werden auch als frei verfügbarer digitaler Rechner („SplenoCalc“) webbasiert zur Verfügung gestellt, wodurch eine exaktere, geschlechterspezifisch und körpergrößenadaptierte Abschätzung der Milzlänge ermöglicht wird [6].

Die Milzgröße nimmt im Alter ab („Altersmilz“), große, voluminöse Milzen präsentieren sich als Splenomegalie bei einer Reihe infektiologischer Erkrankungen (klassisch bei der infektiösen Mononukleose), bei der Sarkoidose und typischerweise bei einer Vielzahl hämatologischer Krankheitsbilder, wie den Leukämien, myeloischen Neoplasien und der eher seltenen systemischen Mastozytose. Die Bestimmung und Wertung der Milzgröße ist ein grundlegendes Puzzleteil auch zur Differenzialdiagnose fokaler Herdbildungen in vergrößerten Milzen.

Nahezu jedes Ultraschallgerät verfügt über einen *Konvex- *und einen *Linearschallkopf*. Bei der praktischen Untersuchung der Milz wird zunächst die Milzlänge mit dem Konvexschallkopf erfasst. Im Anschluss wird die Milz auf fokale Befunde hin analysiert. Dies erfolgt durch ein langsames (!), fächerförmiges Kippen des Konvexschallkopfes in beide Richtungen, meist in Höhe des hohen Interkostalschnitts links. Die Atemposition muss individuell ermittelt werden. Wird der Schallkopf weit kaudal aufgesetzt, ist eine Einatmung möglich, ansonsten ist durch das „Vorhangsphänomen“ der Lunge bei tiefer Inspiration die Sicht oft schlechter als bei normaler Atmung. Auch eine Untersuchung im Sitzen von dorsal kann erwogen werden, gerade zur verbesserten Darstellung fokaler Läsionen im Zwerchfelldom. Sollten fokale oder diffuse Veränderungen (inhomogenes Milzparenchymmuster) auffallen, ist eine zusätzliche Untersuchung mit einem hochfrequenten Linearschallkopf (z. B. 9–4 MHz) vorzunehmen, als eine wichtige Ergänzung, um gerade multiple, kleine fokale Veränderungen nicht zu übersehen (Abb. 1b). Nicht immer kann die Milz komplett eingesehen werden. Dies ist eine relevante Limitation. Hier ist im Einzelfall eine Übersichtsbildgebung zu veranlassen.

Die Detektion fokaler Läsionen erfolgt durch langsames, fächerförmiges Kippen des Konvexschallkopfes

Als Abschluss des B‑US der Milz wird perisplenisch mit dem Konvexschallkopf noch nach Nebenmilzen und auch nach Milzhiluslymphknoten und vaskulären Umgehungskreisläufen gesucht.

Die *farbkodierte Dopplersonographie (FKDS)* ist hilfreich zur Darstellung der zuführenden Milzgefäße und zur Erkennung von Aneurysmata der Milzarterie. Bei der Differenzialdiagnostik für Milzläsionen ist die FKDS vor allem für einen „ersten Eindruck“ zur Vaskularisation der Läsionen hilfreich. So fallen etwa gerade Hämangiome/Splenome durch ein deutlich gesteigertes Gefäßmuster bereits in der FKDS auf und können als solide vitale Läsionen klassifiziert werden. Milzinfarkte und Milzhämatome, seien sie subkapsulär oder intraparenchymal gelegen, sowie Milzabszesse sind durch einen fehlenden Nachweis von Flusssignalen in der FKDS charakterisiert. Für die sichere Unterscheidung von perfundiertem und nichtperfundiertem Milzgewebe zeigt die FKDS - besonders bei unzureichender Geräteeinstellung - Limitationen und wird daher im klinischen Alltag häufig durch die kontrastverstärkte Sonographie (= „contrast-enhanced ultrasound“ oder „CEUS“) ersetzt. Neue Dopplerverfahren (Breitband-Doppler bzw. spezielle Powerdoppler) können sehr hilfreich sein, um größere und mittelgroße Milzinfarkte als solche besser zu identifizieren. Im Pulsed-wave-Doppler (pw-Doppler) lassen sich zudem venöse von arteriellen Spektren innerhalb von Milzherden unterscheiden.

Mit der CEUS kann die Perfusion der Milz in Echtzeit verfolgt werden

Eine wesentliche diagnostische Weiterentwicklung auch zur Dignitätseinschätzung fokaler Milzherde stellt die *Kontrastmittelsonographie (CEUS) *dar. Bekanntermaßen hat die CEUS die Diagnostik der fokalen inzidentellen Leberherde revolutioniert und ist hier mittlerweile diagnostischer Standard. Bei der CEUS werden wenige Milliliter einer Mikrobläschensuspension (SF-6) venös injiziert. Notwendig ist seitens der Ultraschallgeräte ein spezielles Kontrastmittel-Preset, das nur manche Mittelklasse- und vor allem Highend-Geräte aufweisen. Diese Programme im Ultraschallgerät arbeiten mit einem niedrigen mechanischen Index, um nicht die empfindlichen Mikrobläschen (ca. 4–8 µg Größe) zu zerstören. Hierdurch kann die Perfusion der Milz – und potenzieller Milzherde– in Echtzeit verfolgt werden. Zudem hilft die CEUS, das Milzparenchym – auch in Form von Nebenmilzen – an jeder beliebigen Stelle im Bauchraum zu diagnostizieren. Das Milzparenchym weist im Vergleich zu anderen parenchymatösen Organen in der CEUS ein arteriell starkes und extrem langanhaltendes Perfusionsverhalten auf, das oft noch nach länger als 5 min nach Injektion nachweisbar ist [14]. Bei Kontrastmittel (KM) -Sonographien anderer Bauchorgane lohnt es sich daher stets, nach Ende der organspezifischen Untersuchung auch die Milz mit zu untersuchen, um in der Spätphase noch eventuelle Infarkte oder Milzläsionen mit einem abweichenden KM-Verhalten als einfach zu erhebenden Zufallsbefund abgrenzen zu können.

Die Nebenwirkungen des Ultraschallkontrastmittels werden in der Literatur mit 1:10.000 angegeben, meist treten Symptome einer anaphylaktoiden Reaktion auf. Die CEUS sollte daher von notfallmedizinisch trainiertem Personal durchgeführt werden, entsprechende Medikamente (H1-Antihistaminikum/Diphenhydramin, Kortison, Adrenalin, Infusion, Sauerstoff) in unmittelbarer Reichweite sein. Grundsätzlich muss der Patient sein schriftliches Einverständnis geben.

Die entscheidende Information zur Differenzialdiagnose von Milzherden in der CEUS ist die Unterscheidung zwischen perfundierten und nichtperfundierten Milzherden. So lassen sich echogene Zysten und vaskuläre Pathologien wie Hämatome, Abszesse und Infarkte schnell und sicher abgrenzen. In einem zweiten Schritt können vitale Milzherde anhand ihres arteriellen Perfusionsverhaltens unterschieden werden, dabei wird zwischen arteriell hyper- bzw. isoperfundierten Milzherden in Abgrenzung zu den in der CEUS arteriell hypoperfundierten Milzherden unterschieden.

### (Kontrastmittel-)sonographisch gesteuerte Milzpunktion

Die sonographisch gesteuerte Milzpunktion wird von vielen Klinikern immer noch als besonders risikobehaftete Punktion wahrgenommen. In der deutschen DEGUM (Deutsche Gesellschaft für Ultraschall in der Medizin)-Multicenterstudie zu ultraschallgesteuerten Punktionen kam es bei insgesamt 63 Milzinterventionen zu zwei relevanten Komplikationen („major complications“, ca. 3,2 %) und zu einer schweren klinischen Blutung, die zu einer Laparotomie führte [7]. Damit war in dieser Studie die Milzbiopsie die risikoreichste abdominelle Punktion, wenngleich sie die geringste Anzahl der eingeschlossenen Punktionslokalisationen ausmachte.

Die Milzbiopsiestudie von Ignee et al. untersuchte 44 Patienten nach sonographischer Milzbiopsie. Nur bei einem Patienten wurde vier Stunden postpunktionell eine mäßig ausgeprägte Blutung festgestellt, die konservativ behandelt werden konnte. Bei einem weiteren, asymptomatischen Patienten fiel bei einer Abdomensonographie, 2 Wochen nach erfolgter Biopsie, zufällig ein Hämatom auf [8]. Von einer britischen Gruppe wird die Komplikationsrate nach 52 Milzbiopsien an 47 Patienten unter Einsatz einer 18-G-Nadel für größere bzw. kleinere Komplikationen mit null bzw. einer Komplikation angegeben, bei einer diagnostischen Erfolgsrate von 90,4 % [9]. Ergänzend muss erwähnt werden, dass die als Alternative zur Biopsie zu diskutierende diagnostisch-therapeutische Splenektomie ebenfalls mit relevanten Komplikationsraten (17,0 %) und sogar Mortalität (1,6 %) assoziiert ist, abgesehen von den infektiologischen Nebeneffekten, gegen die mit Impfungen vorgebeugt wird [10, 11].

Die sonographisch gesteuerte Punktion ist die primär anzuwendende Methode zur Gewebegewinnung

Sollte eine Läsion sonographisch gut einsehbar sein und die Atemcompliance eine gute Kooperation patientenseitig ermöglichen, stellt die sonographisch gesteuerte Punktion die primär anzuwendende Methode zur Gewebegewinnung für die histopathologische Analyse dar [2].

**Figure 2:**
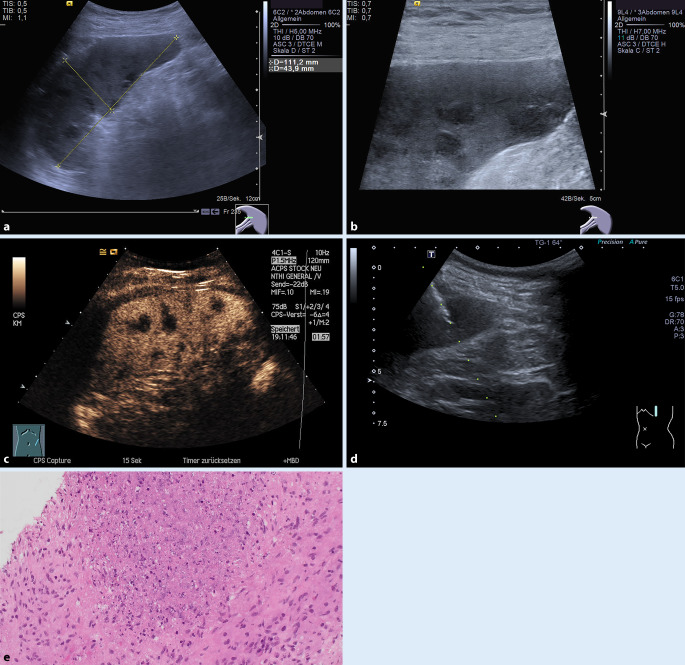

Der Einsatz von Ultraschallpunktionsaufsätzen ermöglicht ein kontrolliertes Vorschieben der Nadel unter Echtzeitbildkontrolle. Bei gleichzeitiger Anwendung der Kontrastmittel (KM)-Sonographie gelingt es auch, bei kleineren Läsionen die Abgrenzbarkeit von Milzparenchymläsionen zu verbessern (Abb. 2a–c).

Grundsätzlich sollte die Indikation zur Milzbiopsie im Tumorboard unter Einbeziehen der interventionellen Radiologie und der Chirurgie gestellt werden.

Bei der Durchführung jeder Milzbiopsie sollten neben Sorgfalt und Respekt vor den Komplikationen stets auch die zeitnahe Verfügbarkeit einer interventionell-radiologischen Einheit bzw. bauchchirurgischer Optionen bedacht werden.

## Differenzialdiagnose und ultraschallbasierter Algorithmus zur Abklärung von Milzherden

In Kenntnis von Anamnese und Klinik sowie der bildgebenden Möglichkeiten der multimodalen Ultraschalldiagnostik gilt es vor allem, benigne von malignen Milzherden hinreichend sicher zu trennen und indifferente Läsionen zu identifizieren, bei denen eine ultraschallgesteuerte Biopsie die Lösung des diagnostischen Dilemmas verspricht.

Bestimmte Ultraschallmodalitäten prädisponieren dabei für bestimmte Fragestellungen. Die häufigen, harmlosen Milzzysten lassen sich meistens bereits im B‑US perfekt und diagnostisch als rundlich-echofreie Läsionen abgrenzen, sie sind meistens kongenital oder posttraumatisch. Für die Infarktdiagnostik bei größeren Milzinfarkten ist neben der B‑US zumindest die FKDS, bei kleineren Milzinfarkten stets die CEUS rasch diagnostisch zielführend [12].

**Figure 3:**
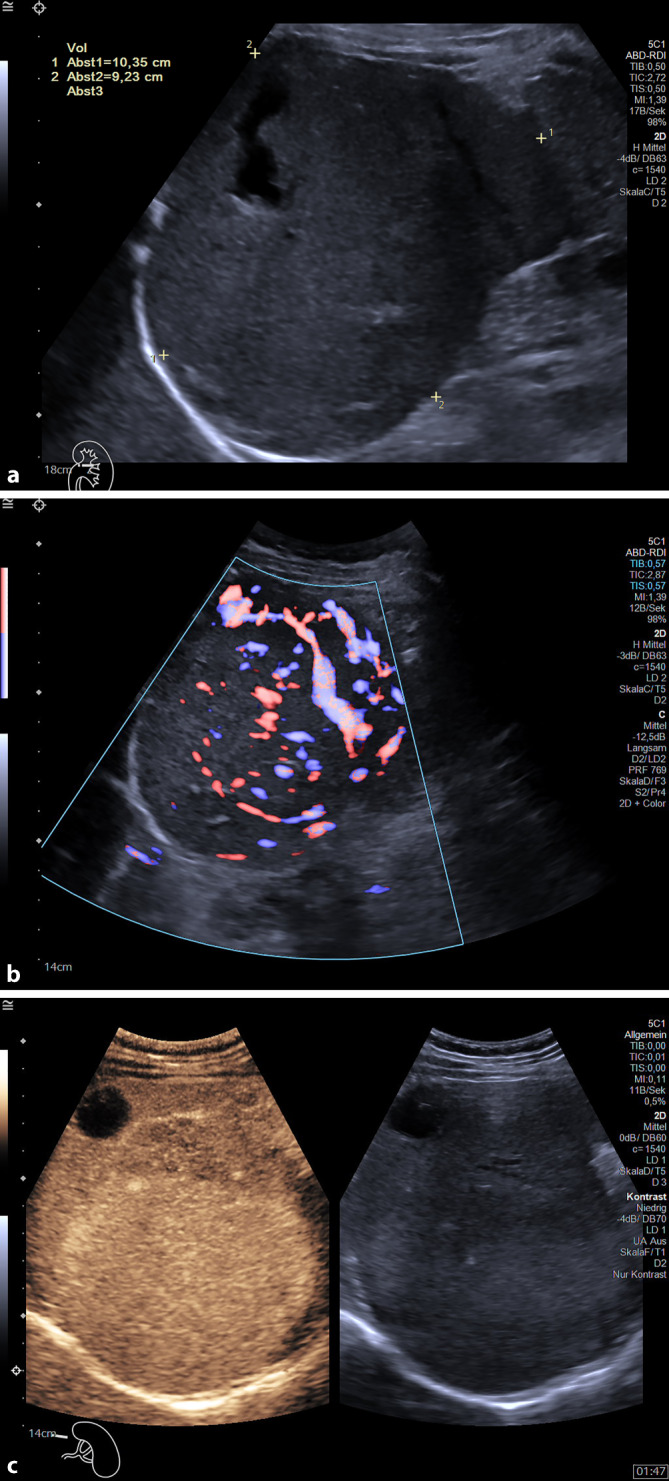

Schwieriger ist die Gruppe der soliden vitalen Milztumoren, wobei grundsätzlich zwischen „Inzidentalomen“ und „Nicht-Inzidentalomen“ differenziert werden muss. Hier muss die Zusammenschau sämtlicher Ultraschallmodalitäten erfolgen, oft sind diese dann diagnostisch für benigne Läsionen, wie die häufigeren Hämangiome/Splenome, die sich im B‑US oft echoreich oder isoechogen präsentieren und sich durch eine deutliche Hypervaskularisation in der FKDS bzw. eine arterielle Hyperperfusion in der CEUS auszeichnen (Abb. 3a–c).

**Figure 4:**
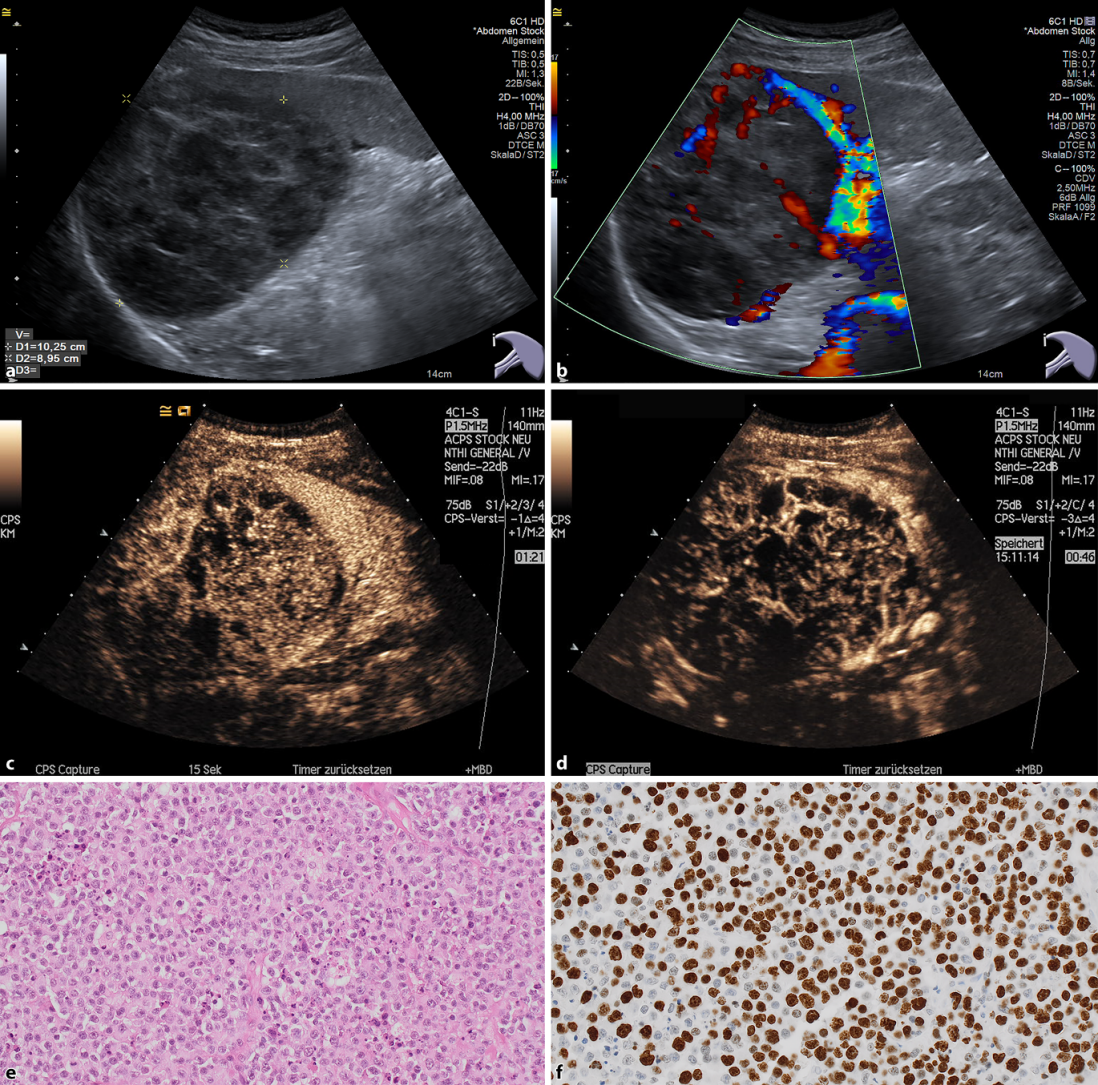

Im Gegensatz dazu imponieren die größeren Lymphomherde oft schwächer perfundiert und weisen zudem ein deutliches Auswaschphänomen auf ([13]; Abb. 4).

**Figure 5:**
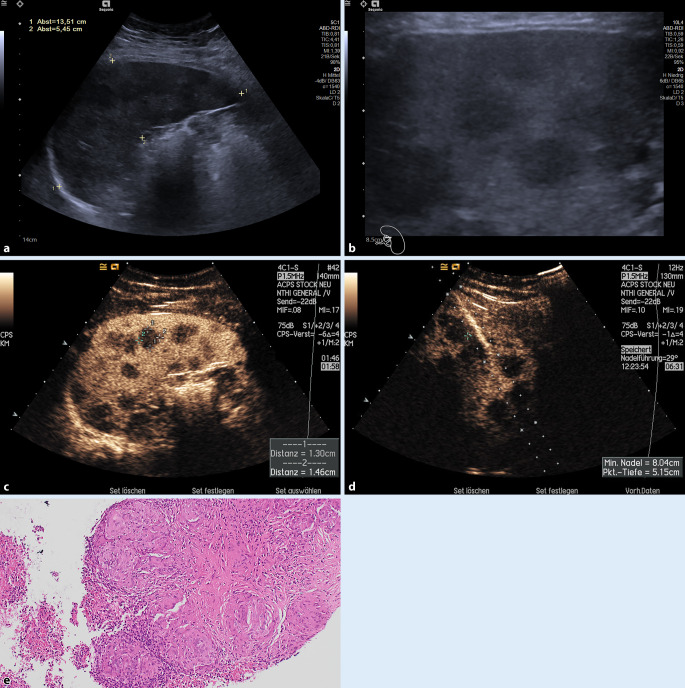

Schwierig ist die Differenzialdiagnose bei multiplen echoarmen Milzherden, die in allen Phasen keine Kontrastmittelaufnahme zeigen. Auch hier ist der klinische Hintergrund entscheidend. Bei diesen Läsionen stellt sich häufig die Differenzialdiagnose zwischen entzündlichen Läsionen, Abszessen, Sarkoidose und Lymphom (Abb. 5). Gelegentlich kann hier die klinische Zusammenschau aller Befunde diagnostisch ausreichend sein. Oft ist gerade in dieser Gruppe in unserem klinischen Alltag eine ultraschallgesteuerte Milzbiopsie von großem diagnostischen Wert und wegweisend für die Therapie [3, 5].

**Figure 6:**
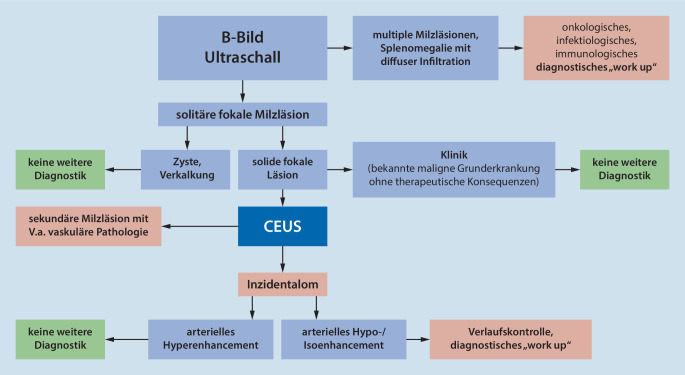

Der Algorithmus zur Abklärung von Milzläsionen fasst unsere Empfehlungen nochmals zusammen (Abb. 6).

## Fazit für die Praxis

- Entscheidend für die Differenzialdiagnose von Milzherden ist die Anamnese, insbesondere die Information über onkologische Vorerkrankungen, um so echte Zufallsbefunde in der Milz besser erkennen zu können.
- Die meisten Zufallsbefunde fokaler Milzläsionen sind benigne.
- Multimodale Ultraschalltechniken und vor allem die kontrastverstärkte Sonographie helfen dabei, zwischen harmlosen benignen Herden (echoreich, hypervaskularisiert) und malignitätsverdächtigen Herdläsionen (echoarm, hypoperfundiert) zu unterscheiden.
- Die ultraschallgesteuerte Milzbiopsie hat zwar eine deutlich höhere Komplikationsrate als die Leberbiopsie, ist bei korrekter und überlegter Indikationsstellung relativ nebenwirkungsarm und oft zielführend.
